# Student characteristics associated with interpersonal skills in medical consultations

**DOI:** 10.1186/s12909-022-03412-9

**Published:** 2022-05-03

**Authors:** Alexandre Bellier, Philippe Chaffanjon, Patrice Morand, Olivier Palombi, Patrice Francois, José Labarère

**Affiliations:** 1 University Grenoble Alpes, School of Medicine, Grenoble, France; 2 Clinical Epidemiology Unit, Grenoble Alpes University Hospital, CS10217, 38043 Grenoble Cedex 09, France; 3 Computational and Mathematical Biology Team, TIMC-IMAG UMR 5525, CNRS, University Grenoble Alpes, Grenoble, France; 4 CIC 1406, INSERM, University Grenoble Alpes, Grenoble, France

**Keywords:** Interpersonal skills, Doctor–patient relationship, Pedagogy, Simulation, Evaluation

## Abstract

**Background:**

The quality of medical care depends on effective physician–patient communication. Interpersonal skills can be improved through teaching, but the determinants are poorly understood. We therefore assessed the factors associated with the interpersonal skills of medical students during simulated medical consultations.

**Methods:**

We conducted a cross-sectional study of fourth-year medical students participating in simulated consultations with standardized patients. Each video-recorded medical consultation was independently assessed by two raters, using a cross-cultural adaptation of the Four Habits Coding Scheme (4-HCS) into French. We then collected information on demographics and education-related characteristics. The relationship between the overall 4-HCS score and student characteristics was modeled using univariable and multivariable linear regression.

**Results:**

Our analytical sample included 165 medical students for analysis. The factors significantly associated with 4-HCS score were gender (β = − 4.8, *p* = 0.011) and completion of an international clinical placement (β = 6.2, *p* = 0.002) or a research laboratory clerkship (β = 6.5, *p* = 0.005). Education-related characteristics, multiple-choice examinations in the first to third preclinical years, and number of medicine or surgery clerkships were not significantly associated with 4-HCS score.

**Conclusions:**

Undergraduate students with higher level of interpersonal skills during video-recorded medical consultations with standardized patients are more likely to be female, to have completed international clinical placement as part of the ERASMUS exchange program or research laboratory clerkship.

**Supplementary Information:**

The online version contains supplementary material available at 10.1186/s12909-022-03412-9.

## Background

Effective physician–patient communication is essential for high-quality medical consultations [1]. Physician communication and interpersonal skills influence patient satisfaction [2], adherence to the care plan [3], and health outcomes [4–6]. Better communication and interpersonal skills would also reduce the likelihood of litigation and malpractice claims [7] and have positive effects on physicians themselves, including greater job satisfaction and decreased risk of burnout [8, 9].

According to the Kalamazoo II report [10], communication and interpersonal skills are two distinct components of effective medical consultations. Communication skills refer to the performance of specific observable tasks*, “that include interviewing to obtain a medical history, explaining a diagnosis and prognosis, giving therapeutic instructions and information needed for informed consent to undergo diagnostic and therapeutic procedures, and providing counselling to motivate participation in therapy or to relieve symptoms*” [10]. Interpersonal skills are “*inherently relational*” and represent “*a set of abilities required to successfully interact and communicate with others, both verbally and non-verbally*”. Interpersonal skills encompass respectful attitude, attention paid to the patient, being personally present in the moment with the patient, interest in patient values and concerns, and real-time adjustment of the relationship [10].

Evidence is accumulating that communication and interpersonal skills can be acquired and improved through teaching and practice-based training for medical students [11–13]. Hence, communication and interpersonal skills training has been advocated as part of the educational curriculum for undergraduate medical students [12, 14–18]. Yet, current evidence on effective approaches to teaching communication skills remains limited [19].

Empirical research has focused on students’ attitude toward communication skills [20–23]. Indeed, attitude toward communication skills is assumed to predict to what extent students will invest in learning these skills and how they will use them when dealing with individual patients [24]. By contrast, few studies examined medical student demographics and education-related characteristics that were associated with observed communication and interpersonal skills. Identifying these factors may help faculty members understand the determinants of medical student communication skills and develop communication and interpersonal skills training programs that take student population characteristics into account [20]. They may include female gender [25–27], academic performance during preclinical years [28], early clinical experience [29–35], and participation in elective programs like international exchange or research laboratory placement.

To deal with this gap in knowledge, the aim of this study was to identify student demographics and education-related characteristics associated with communication and interpersonal skills assessed throughout medical consultations with standardized patients using the Four Habits Coding Scheme (4-HCS). Our primary research hypothesis was a gender difference in 4-HCS scores, consistent with literature. The secondary hypotheses were that 1) better academic performance during preclinical years reflected stronger student motivation and subsequent higher level of communication task fulfillment during medical consultations; 2) advancing clinical experience was associated with higher 4-HCS scores; and 3) participation in elective international exchange or research programs contributed to student interpersonal skills.

## Methods

### Study design

As part of a cross-sectional study [36], we analyzed the original data from video-recorded medical consultations with standardized patients at the University of Grenoble Alpes School of Medicine, France. The present manuscript complies with the STrengthening the Reporting of OBservational studies in Epidemiology (STROBE) guidelines [37].

### Participants

Students who were registered in the fourth year of the medical curriculum were eligible to participate in the project if they passed the medical college admission test. Students were excluded from the present study if they were unavailable at the time of the simulated medical consultations because of leave for academic or personal reason, had been transferred from another school of medicine during their curriculum, or had applied without taking the medical college admission test.

Participants from two consecutive academic years were recruited from October 2016 to December 2016 and from February to April 2018, respectively. Participants were recruited on a voluntary basis.

### Consultations with standardized patients

Each student completed a single video-recorded medical consultation with a standardized patient. For this purpose, seven medical consultation case scenarios were developed, based on the basic medical interview tasks that comprised the 4-HCS. For each medical consultation case scenario, seven clinical problems and seven profiles of portrayals were created according to personality type or character trait (Appendix 1).

The standardized patients consisted of 20 actors recruited at the Department of Performing Arts in Grenoble Alpes University. To ensure consistency in standardized patients, the 20 actors attended a two-hour session with provision of oral and written detailed instructions by a clinician for each medical consultation case scenario. The actors were then trained by their improvisation instructor to portray one to two cases and the profiles of portrayals were chosen from the list just before the consultation. All consultations were video-recorded and videos were stored in a secure cloud-database for subsequent remote access.

### 4-HCS scoring

Each video-recorded medical consultation was independently assessed by two raters, using the cross-cultural adaptation of the 4-HCS into French [36]. The 4-HCS is a standardized instrument designed to assess 23 basic medical interview tasks that are organized within four dimensions, including *Invest in the beginning* (six items), *Elicit the patient’s perspective* (three items), *Demonstrate empathy* (four items), and *Invest in the end* (ten items) [38]. The 4-HCS overall score is computed by summing ratings for the individual items, ranging from 23 (i.e., less effective) to 115 (i.e., more effective). The French version of the 4-HCS previously demonstrated satisfactory internal consistency and acceptable reliability with two independent raters [36]. Cronbach’s alpha was 0.94 for the 4-HCS overall score and intra-class correlation coefficient estimates based on average ratings were 0.82 and 0.91 for inter- and intra-rater reliability, respectively [36].

The two raters were a full professor of medicine and a resident in medicine, both with experience in simulation and teaching communication and interpersonal skills. They performed the assessments at home, usually in sessions of up to 2 consecutive hours. Data were captured using an online form in which the completion of each item of the 4-HCS was mandatory, so there could be no missing data. Because individual ratings were deemed unreliable [36], each student was assigned the average 4-HCS score from two independent raters.

### Data collection

Information on demographics and education-related characteristics was electronically retrieved from databases maintained by the University of Grenoble Alpes School of Medicine. This included age, gender, number of medical college admission test attempts, the total score achieved in the basic science multiple-choice examinations for each of the first- to third-year academic class (range, 0–100), the number of internal medicine, surgery, pediatrics, psychiatry, as well as obstetrics and gynecology clerkship rotations completed, international clinical placement as part of the ERASMUS (EuRopean community Action Scheme for the Mobility of University Students) exchange program [39], and research laboratory placement. For students who took any examination more than once, only the score obtained at the first attempt was analyzed. ERASMUS and research laboratory placements were both elective. ERASMUS placement was conditional to foreign language level certification and the selection process was based on the grade point average achieved in second and third preclinical years. The pre-requisite for research laboratory placement was the validation of two elective disciplinary research courses and formal training on how conducting a research project. Students did not undergo Objective Structured Clinical Examinations (OSCEs) during the first three preclinical years.

### Statistical methods

Summary statistics for the 4-HCS overall score were reported as means along with standard deviations (SDs). To quantify the univariable associations between medical student characteristics and 4-HCS score, crude (unadjusted) β regression coefficient point estimates along with 95% confidence intervals were derived from straight-line linear regression analysis for continuous dependent variable. β regression coefficients quantified the mean change in 4-HCS score for each independent variable category relative to the reference, with null value equal to 0. Adjusted β regression coefficients were derived from multiple linear regression analysis, using the same approach. β regression coefficients were adjusted for international clinical placement, research laboratory placement, and gender. All first-order interactions involving independent variables included in multiple linear regression model were tested for statistical significance.

Because medical consultation case scenarios were randomly assigned, we did not expect heterogeneity in 4-HCS overall scores according to medical problems and profiles of portrayals (Table S1). In unplanned exploratory analysis, we examined the univariable associations for each 4-HCS subscale score with baseline student characteristics.

Two-sided *p*-values of < 0.05 were considered statistically significant. All analyses were performed using RStudio (Version 1.0.143©).

### Ethical considerations

After appropriate written information was delivered, each student signed an individual consent form regarding participation in the research and image rights. An institutional review board (*Comité d’Ethique du Centre d’Investigation Clinique de Clermont-Ferrand*, IRB 00005891) reviewed and approved the study protocol and the information form prior to study initiation. All data were de-identified before analysis.

## Results

### Study sample

**Fig. 1:**
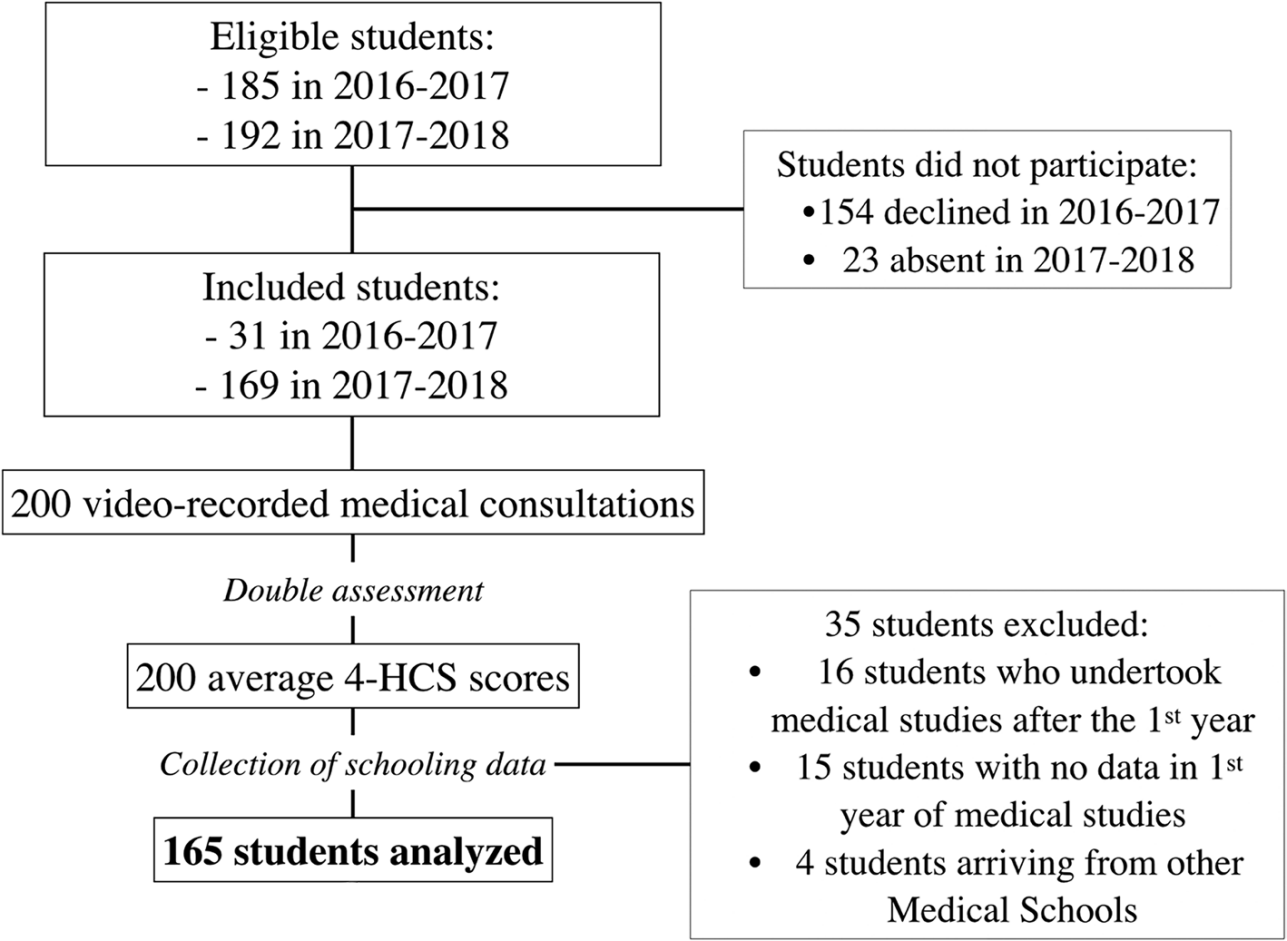
Flowchart of the study

A total of 200 fourth-year medical students (31 and 169 in the 2016–2017 and 2017–2018 academic year, respectively) completed video-recorded consultations with standardized patients. After omitting 35 students according to the exclusion criteria (Fig. 1), our analytic sample consisted of 165 video-recorded medical consultations with standardized patients. The vast majority of participants were female (67% [111/165]) and the mean age was 21.8 years (SD = 1.4). Most students were admitted to the school of medicine after a second attempt (54% [89/165]) and the median examination grades ranged from 73.6 in the first year to 66.8 in the third year. The median numbers of medicine and surgery clerkships were 3 (range, 0–5) and 1 (range, 0–4), respectively. Overall, 47 students (28.5%) had completed a research internship and 29 had participated in the ERASMUS international exchange program (17.6%). Female students had better examination grades, fewer research laboratory placement and comparable ERASMUS and hospital medical placement rates than their male counterparts (Table S2).

### 4-HCS scoring

The mean 4-HCS overall score was 75.4 (SD = 11.8) for all participants. Intra-class correlation coefficient estimate for inter-rater reliability based on individual ratings was 0.76. In univariable analysis, female gender, completion of a research laboratory placement, and international clinical placement as part of the ERASMUS exchange program were associated with a higher 4-HCS overall score (Table 1). These associations remained significant in multivariable analysis (Table 2). Our study failed to show any significant relationship between 4-HCS overall score and the number of medical college admission test attempts, the total score achieved in basic science multiple-choice examinations, and the number of internal medicine and surgery clerkship rotations completed in both univariable and multivariable analyses (Tables 1 and 2). No significant first-order interaction involving independent variables included in multiple linear regression was found.

**Table 1:** Univariable associations of 4-HCS overall score with demographic and education-related characteristics for undergraduate medical students (*n* = 165)

Characteristics		4-HCS^b^ Mean (SD)	β regression coefficient (95%CI)^a^	
			Unadjusted	***p***
**Gender**	Men (*n* = 54)	72.4 (13.5)	−4.4 (− 8.2;-0.6) Reference	0.024
	Women (*n* = 111)	76.8 (10.6)		
**Research laboratory clerkship**	Yes (*n* = 29)	79.6 (8.7)	5.1 (0.4;9.9)	0.032
	No (*n* = 136)	74.5 (12.2)	Reference	
**International clinical placement**	Yes (*n =* 47)	79.4 (10.4)	5.6 (1.7;9.5)	0.006
	No (*n* = 118)	73.8 (11.9)	Reference	
**No. of attempts at MCAT**	1 (*n* = 79)	76.7 (11.2)	2.5 (− 1.1;6.1)	0.180
	> 1 (*n* = 86)	74.2 (12.2)	Reference	
**First year examination score**	1st quartile [68.3–71.2]	73.0 (12.7)	Reference	
	2nd quartile [71.3–73.5]	78.5 (11.5)	5.6 (0.4;10.7)	
	3rd quartile [73.6–75.8]	76.6 (11.1)	3.7 (−1.2;8.5)	0.866
	4th quartile [75.9–87.6]	74.2 (11.1)	1.3 (− 3.6;6.2)	
**Second year examination score**	1st quartile [51.8–66.4]	72.9 (11.4)	Reference	
	2nd quartile [66.5–70.9]	76.5 (11.7)	3.6 (−1.5;8.7)	
	3rd quartile [71.0–75.3]	77.0 (10.0)	4.2 (− 0.9;9.3)	0.292
	4th quartile [75.4–87.0]	74.9 (10.6)	2.0 (−3.2;7.3)	
**Third year examination score**	1st quartile [38.0–62.0]	73.3 (15.2)	Reference	
	2nd quartile [62.1–66.7]	75.7 (10.8)	2.4 (−2.6;7.5)	
	3rd quartile [66.8–71.7]	77.1 (9.9)	3.7 (−1.5;8.9)	0.302
	4th quartile [71.8–84.3]	75.3 (10.7)	2.0 (−3.2;7.2)	
**Medicine clerkship**	No (*n* = 2)	85.1 (2.3)	Reference	
	One (*n* = 8)	76.9 (10.3)	−8.3 (−26.5;10.0)	
	Two (*n* = 35)	77.6 (11.5)	−7.5 (−24.3;9.2)	
	Three (*n* = 75)	75.0 (11.8)	−10.2 (− 26.7;6.4)	0.038
	Four (*n* = 40)	74.8 (11.6)	−10.3 (−27.0;6.4)	
	Five (*n* = 5)	64.5 (15.0)	−20.7 (−40.0;-1.4)	
**Surgery clerkship**	No (*n* = 28)	74.1 (13.5)	Reference	
	One (*n* = 80)	77.0 (9.8)	2.8 (−2.3;8.0)	
	Two (*n* = 46)	74.0 (13.1)	−0.1 (−5;7;5;5)	0.478
	Three (*n* = 10)	71.5 (14.6)	−2.7 (−11.2;5.9)	
	Four (*n* = 1)	80.8	6.6 (−17.1;30.3)	

*Abbreviations*: *MCAT* Medical College Admission Test, *CI* Confidence Interval, *4-HCS* 4 Habits Coding Scheme, *SD* Standard Deviation

^a^ β regression coefficients quantified the change in 4-HCS score for each independent variable category relative to the reference, with null value equal to 0

^b^ 4-HCS range: [23–115]

**Table 2:** Multivariable associations of 4-HCS overall score with demographic and education-related characteristics for undergraduate medical students (*n* = 165)

Characteristics		4-HCS^b^ Mean (SD)	β regression coefficient (95%CI)^a^	
			Adjusted^c^	***p***
**Gender**	Men (*n* = 54)	72.4 (13.5)	−4.8 (−8.4;-1.1)	0.011
	Women (*n* = 111)	76.8 (10.6)	Reference	
**Research laboratory clerkship**	Yes (*n* = 29)	79.6 (8.7)	6.5 (1.9;11.0)	0.005
	No (*n* = 136)	74.5 (12.2)	Reference	
**International clinical placement**	Yes (*n =* 47)	79.4 (10.4)	6.2 (2.3;10.0)	0.002
	No (*n* = 118)	73.8 (11.9)	Reference	
**No. of attempts at MCAT**	1 (*n* = 79)	76.7 (11.2)	1.3 (−2.3;4.9)	0.480
	> 1 (*n* = 86)	74.2 (12.2)	Reference	
**First year examination score**	1st quartile [68.3–71.2]	73.0 (12.7)	Reference	
	2nd quartile [71.3–73.5]	78.5 (11.5)	2.5 (1.1;10.9)	
	3rd quartile [73.6–75.8]	76.6 (11.1)	2.4 (−1.1;8.4)	0.717
	4th quartile [75.9–87.6]	74.2 (11.1)	2.4 (−4.5;5.0)	
**Second year examination score**	1st quartile [51.8–66.4]	72.9 (11.4)	Reference	
	2nd quartile [66.5–70.9]	76.5 (11.7)	3.9 (−0.9;8.7)	
	3rd quartile [71.0–75.3]	77.0 (10.0)	1.5 (−3.5;6.5)	0.851
	4th quartile [75.4–87.0]	74.9 (10.6)	2.6 (−4.7;5.4)	
**Third year examination score**	1st quartile [38.0–62.0]	73.3 (15.2)	Reference	
	2nd quartile [62.1–66.7]	75.7 (10.8)	1.5 (−3.3;6.4)	
	3rd quartile [66.8–71.7]	77.1 (9.9)	0.3 (−4.9;5.5)	0.916
	4th quartile [71.8–84.3]	75.3 (10.7)	0.2 (−4.9;5.3)	
**Medicine clerkship**	No (*n* = 2)	85.1 (2.3)	Reference	
	One (*n* = 8)	76.9 (10.3)	−12.0 (−29.7;5.7)	
	Two (*n* = 35)	77.6 (11.5)	−8.4 (−24.6;7.8)	
	Three (*n* = 75)	75.0 (11.8)	−10.5 (−26.6;5.5)	0.256
	Four (*n* = 40)	74.8 (11.6)	−10.0 (−26.5;6.4)	
	Five (*n* = 5)	64.5 (15.0)	−18.1 (−36.9;0.8)	
**Surgery clerkship**	No (*n* = 28)	74.1 (13.5)	Reference	
	One (*n* = 80)	77.0 (9.8)	4.1 (−0.8;9.1)	
	Two (*n* = 46)	74.0 (13.1)	3.0 (−2.5;8.6)	0.403
	Three (*n* = 10)	71.5 (14.6)	3.1 (−5.5;11.6)	
	Four (*n* = 1)	80.8	9.9 (−12.7;32.5)	

*Abbreviations*: *MCAT* Medical College Admission Test, *CI* Confidence Interval, *4-HCS* 4 Habits Coding Scheme, *SD* Standard Deviation

^a^ β regression coefficients quantified the change in 4-HCS score for each independent variable category relative to the reference, with null value equal to 0

^b^ 4-HCS range: [23–115]

^c^ Adjusted for international clinical placement, research laboratory clerkship and gender

No evidence of inconsistencies in univariable associations of baseline student characteristics was observed across 4-HCS subscale scores (Table S3), although no formal conclusions could be drawn from these exploratory analyses.

## Discussion

This study provides important insight into undergraduate student characteristics that are potential determinants of communication performance observed during video-recorded medical consultations with standardized patients. The characteristics independently associated with 4-HCS overall score included female gender, completion of a research laboratory placement, and international clinical placement. The number of medical college admission test attempts, basic science examination scores, and the number of medicine or surgery clerkship rotations completed were not predictive of 4-HCS overall scores.

To our knowledge, limited data exist on the relationship between examination scores or number of medical college admission test attempts and communication skills for medical students. A previous study reported a weak but significant correlation between academic examination scores undertaken over the first 2 years of medical school and subsequent OSCEs [28], which constitute an alternative approach for assessing student communication and interpersonal skills [40]. Our study failed to show any significant association between basic science multiple-choice examination scores and subsequent 4-HCS overall score. Although we cannot exclude that our study was underpowered to detect a significant correlation, this negative finding likely reflects the fact that communication and interpersonal skills are independent concepts from technical skills evaluated throughout standardized examinations. This latter observation supports the incorporation of communication skills training and assessment as part of the medical curriculum beside standardized examinations on basic science or clinical knowledge.

International clinical placement as part of the ERASMUS exchange program was associated with higher 4-HCS overall scores in our study. Previous studies have suggested that international clinical placement would provide students with the opportunity to develop communication skills [41]. Yet, the potential for self-selection bias cannot be excluded, reflecting the fact that students with a higher level of communication skills at baseline were selected for international clinical placement and subsequently yielded better communication performance during medical consultations with standardized patients.

Research laboratory placement was a local initiative at our medical school and therefore the report of similar findings in the literature is unlikely. We hypothesize that the benefits of research laboratory placement include the development of teamwork and interpersonal skills. As part of their training on how conducting a research project, students are strongly recommended to collaborate with multidisciplinary teams including laboratory technicians, clinical research associates and supervisors. Moreover, the final evaluation of research laboratory clerkship includes among others assessment of collaborative relationships by the project supervisor, a fact that may foster student interpersonal skills.

Surprisingly, we did not observe any significant relationship between the number of internal medicine or surgery clerkship rotations completed and 4-HCS scores. Basically, we expected that interpersonal skills improved with hospital clinical experience. The scant results from the literature suggest an increase in interpersonal skills with an early immersion in an internship [29]. Yet a decline of empathy during the medical curriculum has been reported by several studies [31–35]. Although speculative, a potential explanation might be that the repetition of tasks, the work overload, and the high emotional load of certain clinical situations lead students pay less attention to interpersonal skills. Hence, advancing clinical experience may result in better accomplishment of communication tasks that is attenuated by decrease in interpersonal skills.

Our finding that female gender was associated with higher 4-HCS overall score is consistent with previous research indicating that female students tend to achieve higher grades in clinical communication tasks than their male counterparts [42].

Krupat et al. found that female medical students were more patient-centered [27]. This difference was observed very early in the medical curriculum and guided the career choice with a greater interest in primary care. These differences can be explained by women’s increased interest in communication and listening in comparison with men [26], particularly in nonverbal communication [43]. Meta-analyses have revealed that consultation time is significantly greater for women than for men and that female physicians showed greater engagement in communication [44, 45]. Beyond communication, it has been shown that female medical students developed more empathy [32]. The choice of different career orientations is reflected in our results, since female medical students tend to have more hospital medical placements and fewer surgery placements. Female students also have significantly better academic results in the majority of disciplines. However, the academic results are not associated with the interpersonal skills measured by the 4-HCS scale. The discrepancy in the numbers of male versus female participants in our study reflected the larger proportion of females among medical students at our university. Reassuringly, the participation rate did not differ between male and female medical students (55% versus 71%, *P* = 0.481).

This study has potential implications for optimizing communication skills training and assessment as part of the medical curriculum. First, objective measures such as multiple-choice examinations do not predict medical student communication performance, and the assessment of communication and interpersonal skills requires specific – although subjective – evaluations, such as consultations with standardized patients. Second, internal medicine clerkship should include other training components than clinical reasoning and technical skills so as to provide students with the opportunity to acquire experience in communication with real patients. Third, male medical students may benefit from specific training, so as to gain the same level of communication skills as their female counterparts. Fourth, future research should examine the role of other important variables, including personality and interpersonal style, in the development of communication and interpersonal skills during medical studies.

The limitations of our study should be acknowledged. First, each student had the opportunity to conduct only one video-recorded consultation, and this may not reflect his / her communication performance in routine practice. Second, we may have omitted some relevant characteristics relating to 4-HCS scoring, including ethnic minority, socioeconomic status, or cognitive factors such as emotional intelligence. Importantly, attitude toward communication skills was not measured in our study, a potential determinant of observed communication. Third, our study was conducted at a single study site and our findings may not apply to other settings or geographical areas. Fourth, between-actor heterogeneity could not be investigated in this study, and we could not exclude that this heterogeneity might partly explain our findings despite random assignment. Fifth, inter-rater reliability (0.76) was lower than previously reported (0.82). This apparent inconsistency likely reflects different computational approaches, although between-study heterogeneity in rater level of experience and random sampling error cannot be excluded. Indeed, inter-rater reliability was quantified using average ratings over two out of four raters in the previous study [36]. Because only two raters participated in the present study, average ratings could not be computed. Reassuringly, intra-class correlation coefficient based on individual ratings in the present study was higher than 0.70, which is indicative of satisfactory inter-rater reliability.

## Conclusions

Through medical consultation simulation exercises and standardized assessment of medical students’ interpersonal skills, we showed that the main factors associated with interpersonal skills were gender and participation in an international clinical placement or a research laboratory clerkship without influence of education-related characteristics or hospital experience. Based on our results, we showed the importance of these simulation exercises with the assessment of nontechnical skills. Indeed, multiple-choice examinations do not predict the level of interpersonal skills, and hospital medicine clerkship does not seem to improve them. It would be interesting to study the decline of empathy, especially among medical students. A major challenge for initial training would be to avoid this decline. It is likely that simulation exercises such as those we have organized will help students realize the importance of these skills and limit the unavoidable decline.

## Supplementary Information

**Additional file 1: Table S1.** Medical consultation case scenarios and profiles of portrayals.

**Additional file 2: Table S2.** Comparison of undergraduate medical student characteristics according to gender (*n* = 165).

**Additional file 3: Table S3.** Exploratory analysis of univariable associations between 4-HCS subscale scores and demographic and education-related characteristics for undergraduate medical students (*n* = 165).

## Data Availability

In accordance with the French guidelines, the data set will be archived for at least 10 years after publication. The data set will be available on Harvard Dataverse public repository: 10.7910/DVN/RHZVKY.

## Abbreviations

4-HCS: Four Habits Coding Scheme
SD: Standard Deviation
