# Supplementary material for: Student characteristics associated with interpersonal skills in medical consultations
Source: BMC Med Educ. 2022 May 3;22:338. doi: 10.1186/s12909-022-03412-9 (PMC9063305; doi:10.1186/s12909-022-03412-9)
Supplement: Supplementary file 1 — Additional file 1: Table S1. Medical consultation case scenarios and profiles of portrayals. [file 12909_2022_3412_MOESM1_ESM.docx]

**Table S1.** Medical consultation case scenarios and profiles of portrayals

| Clinical problems | Profiles of portrayals |
| --- | --- |
| Significant fatigue with a decrease in physical and mental performance in the context of a depressive syndrome | Reserved and shy patient with a tendency to retain information |
| History of hemorrhage during dental extraction with genetic background in the context of a coagulation disorder | Disorganized logorrheic patient |
| New-onset axillary adenopathy after cat scratch in the context of an infectious disease | Anxious and apprehensive patient, taking notes |
| Episode of tachycardia and moderate exercise-induced asthma in a young athlete | Patient demanding and in a hurry, sometimes aggressive |
| Incidental finding of microscopic hematuria on urine dipstick with a history of macroscopic hematuria | Curious and knowledgeable patient |
| Right chest pain with isolated dry cough of recent onset and history of rheumatism in a young woman | Patient with limited understanding |
| Significant weight loss due to recent-onset diarrhea in an infectious situation. | Minimizing and carefree patient |
