# Supplementary material for: Student characteristics associated with interpersonal skills in medical consultations
Source: BMC Med Educ. 2022 May 3;22:338. doi: 10.1186/s12909-022-03412-9 (PMC9063305; doi:10.1186/s12909-022-03412-9)
Supplement: Supplementary file 2 — Additional file 2: Table S2. Comparison of undergraduate medical student characteristics according to gender (n = 165). [file 12909_2022_3412_MOESM2_ESM.docx]

**Table S2.** Comparison of undergraduate medical student characteristics according to gender (*n* = 165).

|  | Gender | | | | | | | |  | |  |
| --- | --- | --- | --- | --- | --- | --- | --- | --- | --- | --- | --- |
| Characteristics | Female  (n = 111) | | | | Male  (n = 54) | | | | *p* | |  |
| Research laboratory clerkship, *n (%)* | | 17 (15.3) | |  | | 12 (22.2) | |  | | 0.028 | |
| International clinical placement, *n (%)* | | 32 (28.8) | |  | | 15 (27.8) | |  | | 0.888 | |
| >1 attempts at MCAT, *n (%)* | | 49 (44.1) | |  | | 30 (55.6) | |  | | 0.170 | |
| First year examination score, median (IQR) | 74.0 (4.8) | |  | | 72.7 (4.6) | |  | | 0.182 | |  |
| Second year examination score, median (IQR) | 71.9 (8.6) | |  | | 68.6 (7.8) | |  | | 0.008 | |  |
| Third year examination score, median (IQR) | 67.6 (10.1) | |  | | 63.7 (8.0) | |  | | 0.012 | |  |
| No. medicine clerkships, median (IQR) | 3 (1) | |  | | 3 (1) | |  | | 0.054 | |  |
| No. Surgery clerkships, median (IQR) | 1 (1) | |  | | 1 (1) | |  | | 0.259 | |  |

Abbreviations: MCAT = Medical College Admission Test, IQR = interquartile range (i.e., 25-75^th^ percentiles)
