# Supplementary material for: Student characteristics associated with interpersonal skills in medical consultations
Source: BMC Med Educ. 2022 May 3;22:338. doi: 10.1186/s12909-022-03412-9 (PMC9063305; doi:10.1186/s12909-022-03412-9)
Supplement: Supplementary file 3 — Additional file 3: Table S3. Exploratory analysis of univariable associations between 4-HCS subscale scores and demographic and education-related characteristics for undergraduate medical students (n = 165). [file 12909_2022_3412_MOESM3_ESM.docx]

**Table S3.** Exploratory analysis of univariable associations between 4-HCS subscale scores and demographic and education-related characteristics for undergraduate medical students (*n* = 165).

|  | Invest in the beginning | | Elicit the patient’s perspective | Demonstrate empathy | | Invest in the end | |
| --- | --- | --- | --- | --- | --- | --- | --- |
| No. items | 6 | 3 | | | 4 | | 10 |
| Subscale score, median (range)* | 61.7 (44.2) | 66.7 (63.3) | | | 68.8 (68.8) | | 67.5 (64.0) |
| Unadjusted β coefficient (95%CI) |  |  | | |  | |  |
| Male gender | -3.3 (-6.2;-0.4) | -1.7 (-6.0;-2.6) | | | -5.4 (-9.7;-1.2) | | -4.1 (-8.0;-0.2) |
| Research laboratory placement | 2.8 (-0.8;6.4) | 4.6 (-0.7;9.9) | | | 4.7 (-0.6;10.0) | | 5.3 (0.5;10.1) |
| International clinical placement | 5.0 (2.1;8.0) | 6.7 (2.3;11.1) | | | 2.3 (-2.1;6.8) | | 5.2 (1.2;9.2) |
| Single attempt at MCAT | 0.9 (-1.9;3.7) | 1.9 (-2.1;6.0) | | | 0.2 (-3.8;4.3) | | 3.7 (0.1;7.3) |
| First year examination score | -0.1 (-0.4;0.3) | -0.1 (-0.6;0.5) | | | 0.1 (-0.5;0.6) | | 0.1 (-0.4;0.6) |
| Second year examination score | 0.0 (-0.2;0.2) | -0.1 (-0.4;0.3) | | | 0.0 (-0.3;0.3) | | 0.3 (0.0;0.6) |
| Third year examination score | 0.0 (-0.2;0.2) | 0.0 (-0.3;0.3) | | | 0.0 (-0.3;0.3) | | 0.3 (0.0;0.5) |
| No. medicine clerkships | -1.6 (-3.1;-0.2) | -4.0 (-6.1;-1.9) | | | -1.3 (-3.5;0.8) | | -1.3 (-3.3;0.6) |
| No. surgery clerkships | -0.4 (-2.1;1.2) | 0.7 (-1.7;3.2) | | | -0.3 (-2.7;2.2) | | -1.4 (-3.6;0.8) |

^*^ 4-HCS subscale ranged from 0 to 100.

Abbreviations: MCAT = Medical College Admission Test, CI = Confidence Interval ; 4-HCS = 4 Habits Coding Scheme
